# Current Strategies for Tumor Photodynamic Therapy Combined With Immunotherapy

**DOI:** 10.3389/fonc.2021.738323

**Published:** 2021-11-17

**Authors:** Jianfeng Hua, Pan Wu, Lu Gan, Zhikun Zhang, Jian He, Liping Zhong, Yongxiang Zhao, Yong Huang

**Affiliations:** ^1^ National Center for International Research of Bio-targeting Theranostics, Guangxi Key Laboratory of Bio-targeting Theranostics, Collaborative Innovation Center for Targeting Tumor Diagnosis and Therapy, Guangxi Talent Highland of Bio-targeting Theranostics, Guangxi Medical University, Nanning, China; ^2^ The First People’s Hospital of Changde City, Changde, China

**Keywords:** photodynamic therapy, immunotherapy, combination, tumor, nanoparticle

## Abstract

Photodynamic therapy (PDT) is a low invasive antitumor therapy with fewer side effects. On the other hand, immunotherapy also has significant clinical applications in the treatment of cancer. Both therapies, on their own, have some limitations and are incapable of meeting the demands of the current cancer treatment. The efficacy of PDT and immunotherapy against tumor metastasis and tumor recurrence may be improved by combination strategies. In this review, we discussed the possibility that PDT could be used to activate immune responses by inducing immunogenic cell death or generating cancer vaccines. Furthermore, we explored the latest advances in PDT antitumor therapy in combination with some immunotherapy such as immune adjuvants, inhibitors of immune suppression, and immune checkpoint blockade.

## Introduction

Cancers are chronologic diseases that seriously threaten human life. Many strategies have been developed for cancer treatment, including chemotherapy, radiotherapy, surgery, and targeted therapy, which are found to be effective for some malignant tumors (1, 2). However, metastasis, recurrence, heterogeneity, resistance to chemotherapy and radiotherapy, and avoidance of immunological surveillance are the most common reasons for cancer treatment failure. Therefore, new therapies with targeted and less invasive features are needed for cancer treatment. In 1903, Tappeiner and Jesionek used white light and eosin to treat skin tumors, setting a photodynamic therapy (PDT) model to treat tumors (3). PDT is a minimally invasive therapy that generates cytotoxic reactive oxygen species (ROS) through a light source, molecular oxygen, and organic macrocycles called photosensitizers (PSs) (4, 5). Currently, nanomaterials are widely used as PS carriers due to their better cytocompatibility, lower cytotoxicity, and excellent tumor targeting compared with traditional small-molecule PSs (6, 7). The enhanced permeability and retention (EPR) effect-based nanomedicine has been widely used for tumor targeting during the past decades. PDT only produces ROS in the tumor after local irradiation with excitation light, making it less invasive and confined. Furthermore, PDT has shown promising results in the diagnosis and treatment of cancers, such as breast, colorectal, and skin cancers, because of the mechanism of action of PSs, which does not cause drug tolerance (8–10). PDT can also induce immunogenic cell death (ICD), promote the release of tumor-associated antigens (TAAs) from tumor cell remnants, and increase the proliferation, activation, and infiltration of antigen-presenting cells and antigen-specific T cells (11–13).

Tumor immunotherapy is an innovative therapy that modulates the immune microenvironment and activates the immune system. It depends on autoimmune functions to kill cancer cells and tumor tissues (14, 15). This approach has the advantage of producing long-term immunological memory effects while causing no harm to normal tissues or cells (16). In recent years, with the discovery of tumor immune checkpoint molecules, such as cytotoxic T lymphocyte (CTL)-associated protein-4 (CTLA-4), programmed cell death protein-1 (PD-1), and its ligand PD-L1, important breakthroughs have been made in the study of antitumor immune mechanisms, and immunotherapy has become a promising tumor treatment (17, 18). Currently, the main strategies in cancer immunotherapy include tumor vaccines, adoptive cellular immunotherapy (ACI), and immune checkpoint blockade (ICB) therapy (19–21). However, immune drugs alone are not effective for all patients. Drug resistance or adverse effects, including skin rashes, itching, diarrhea, pneumonia, and thyroid malfunction, may occur in some people (22). In addition, immune side effects, including the cascade of inflammatory mediators, hematopoietic system dysfunction, and organ toxicity, also limit the optimization of immunotherapy methods (23–25). Therefore, it is needed to develop appropriate combined cancer therapies to enhance the effectiveness of immunotherapy and to reduce side effects. It has been revealed that combining PDT with antitumor immunotherapy not only can improve the PDT-induced antitumor immune response but also can promote the proliferation and activation of immune memory cells, inhibit tumor metastasis, and prevent tumor recurrence (26, 27).

In this review, we explain the principles and damage mechanisms of PDT and discuss the immune response induced by PDT. We also summarize the combined treatment strategies of PDT and some immunotherapies, such as immune adjuvants, inhibitors of immune suppression, and ICB for cancer. We believe that this combination therapy strategy will be further developed and functionalized to meet the application in biomedicine, thereby making remarkable contributions to human health.

## The Principle and Damage Mechanism of Photodynamic Therapy

**Figure 1:**
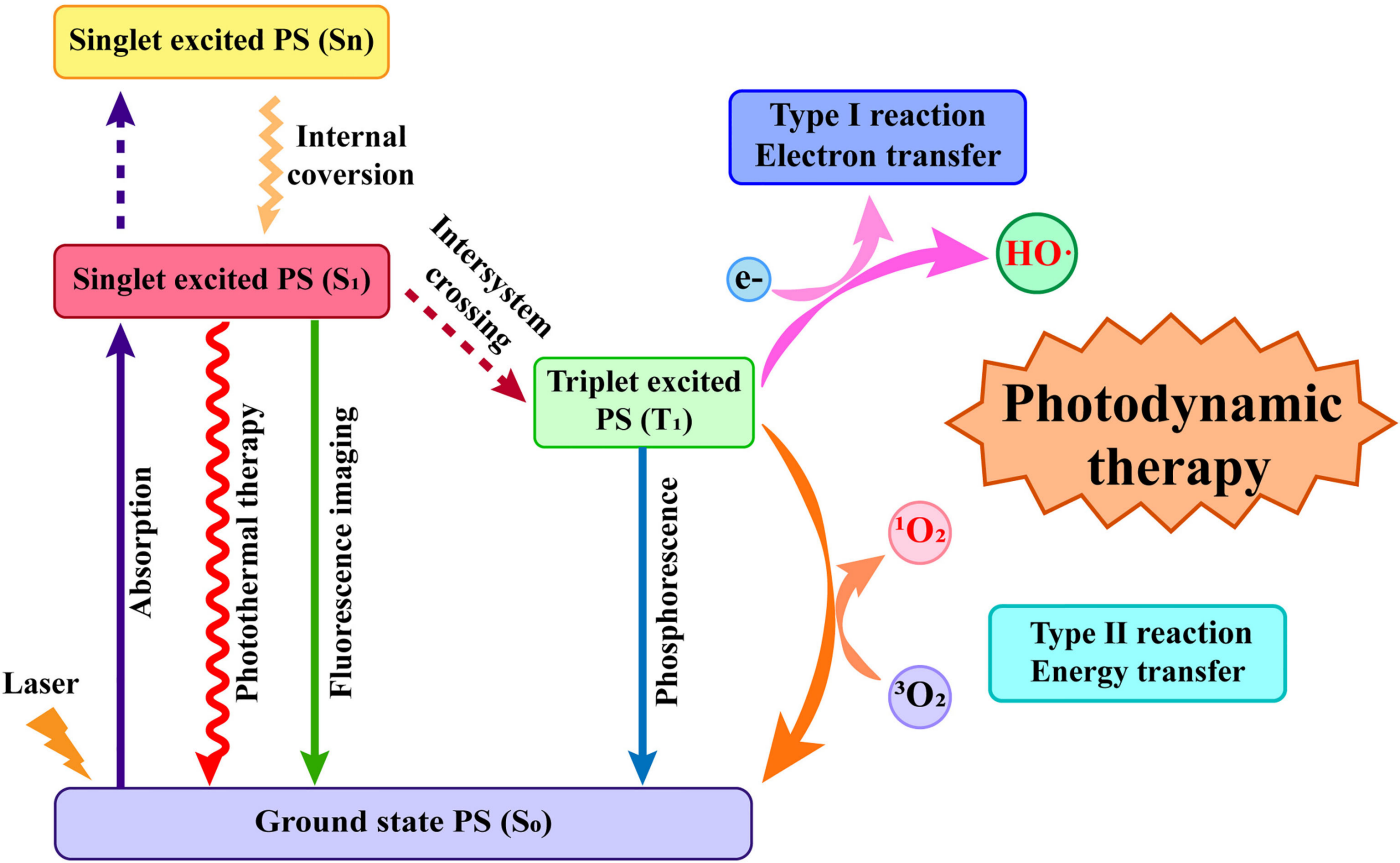
The photophysical mechanism and the two classic types of photodynamic therapy (PDT). The photosensitizer absorbs light energy to jump from the ground state to the excited state, which then leads to a long-lived excited triplet state through intersystem crossing and finally generate reactive oxygen species through type I reaction or type II reaction.

The exact mechanism of PDT has not yet been elucidated. However, the widely accepted theory is based on photophysical principles and guided by the Jablonski Diagram. The Jablonski Diagram clarifies the different electronic states of molecules and the process of their transitions, which are considered as the basic principles for designing phototherapeutic reagents (28, 29). As shown in **Figure 1**, PS is excited by light irradiation, and the electronic state changes from the ground state (S_0_) to the singlet excited state (S_n_) and then relaxes to the lowest energy level of the singlet excited state (S_1_) through internal conversion (IC). The PS at the S_1_ state can consume energy in three ways, resulting in diverse outcomes (30, 31). 1). The molecules at the S_1_ state emit a photon with a longer wavelength to S_0_. The photon emission process can be applied to fluorescence imaging. 2) S_1_ state molecules release heat by colliding with each other and relax to the S_0_ state in a non-radiative manner, which is often used in photothermal therapy. 3) The PS at the S_1_ state transitions to the lowest energy level of the triplet state (T_1_) through intersystem crossing (ISC) (32–34). The molecules at the T_1_ state can relax to the S_0_ state by emitting photons. This phenomenon is called phosphorescence. In addition, molecules in the T_1_ state can also perform two different types of PDT by generating free radicals or singlet oxygen (35–37). In type I reaction, T_1_ state chemicals form free radicals by directly interacting with endogenous substrates, such as cell membranes or biological macromolecules, and subsequently react with oxygen to produce ROS (38). In the type II reaction, T_1_ state molecules directly transfer energy to oxygen molecules in the surrounding environment to generate singlet oxygen, which may oxidize the macromolecular cellular components, resulting in cellular death through either apoptosis or necrosis (39, 40). However, it should be noted that most PSs exert their antitumor effects by causing cell damage through the generation of singlet oxygen from the type II reaction (41). Singlet oxygen can act on protein sulfhydryl and amino groups to denature proteins and reduce enzymatic activity in cells (42, 43). Singlet oxygen can also alter the structure and function of cell membranes, mitochondrial membranes, and DNA molecules (44, 45).

**Figure 2:**
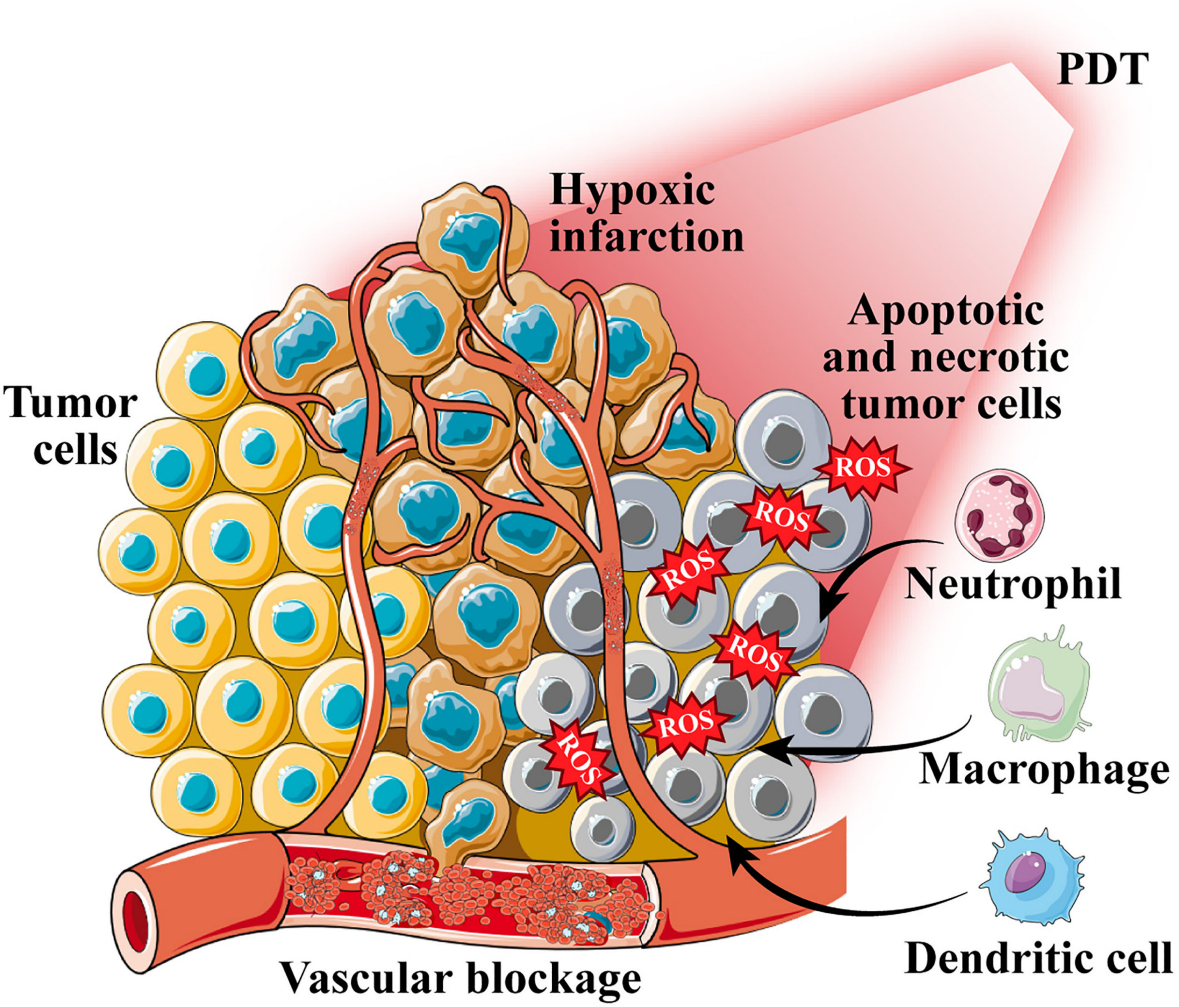
The tumor damage mechanism caused by photodynamic therapy (PDT). PDT generates reactive oxygen species (ROS) to induce cell apoptosis and necrosis; photosensitizers target the vascular system to form thrombi, causing hypoxic infarction of tumor tissues; apoptotic and necrotic tumor cells recruit a variety of white blood cells.

The tumor damage mechanism caused by PDT mainly includes the following types. 1) Direct killing effect of ROS on tumor cells, including apoptosis, necrosis, and autophagy. 2) PSs target the vascular system to form thrombi, causing hypoxic infarction of tumor tissues. 3) Tumor cells that undergo apoptosis or necrosis release inflammatory factors, which trigger an inflammatory response that leads to an antitumor immune response (46–48) (**Figure 2**). It is worth mentioning that apoptosis, autophagy, and cell cycle arrest after PDT may occur simultaneously during a single treatment session. Sasnauskiene et al. (49) found that the degree of oxidative stress damage to cells is dose-dependent. Cells showed increased autophagy and cell cycle arrest but no apoptosis when the cytotoxic dose was increased to 50%. However, the cells displayed significant apoptosis, autophagy, and cell cycle arrest when the cytotoxic dose was greater than 70%. The damage to blood vessels by PDT is based on the characteristics of tumor tissue with wide vascular gaps and poor integrity, which are conducive to PS aggregation (50). After photoactivation, PS enrichment in tumor vascular endothelial cells causes many physiological responses, including platelet aggregation and vasoconstriction, which lead to tumor vascular blockage, ischemia, and hypoxia (51, 52). The direct ablation effect of PDT on tumor cells also releases inflammatory mediators, thereby recruiting a variety of white blood cells, such as neutrophils, macrophages, and dendritic cells (DCs) (53). These white blood cells will then activate the immune cascade to further suppress the tumor (54, 55).

## Photodynamic Therapy and Antitumor Immune Response

### Photodynamic Therapy-Mediated Immunogenic Cell Death

The current elaboration on the mechanism of PDT antitumor immune response tends to focus on PDT-induced oxidative stress in tumor cells, causing ICD and release of TAAs and damage-associated molecular patterns (DAMPs) (56, 57). ICD is a specific cell death mode that translocates calreticulin (CRT) to the cell surface and releases high mobility group box 1 protein (HMGB1), adenosine triphosphate (ATP), and heat shock proteins (HSPs) to the extracellular surface (58, 59). Inflammation-related signaling pathways, the release of immune-related cytokines, neutrophil infiltration, and the complement cascade are all triggered by these DAMPs (60, 61). DCs are specialized antigen-presenting cells that link the innate immune response to the adaptive immune response, taking up TAAs, binding DAMPs through pattern recognition receptors (PRRs), and processing antigens as they migrate to lymph nodes and mature. The antigens are presented to T cells for proliferation and differentiation into CTLs, which exert antitumor immune effects (62–64).

**Figure 3:**
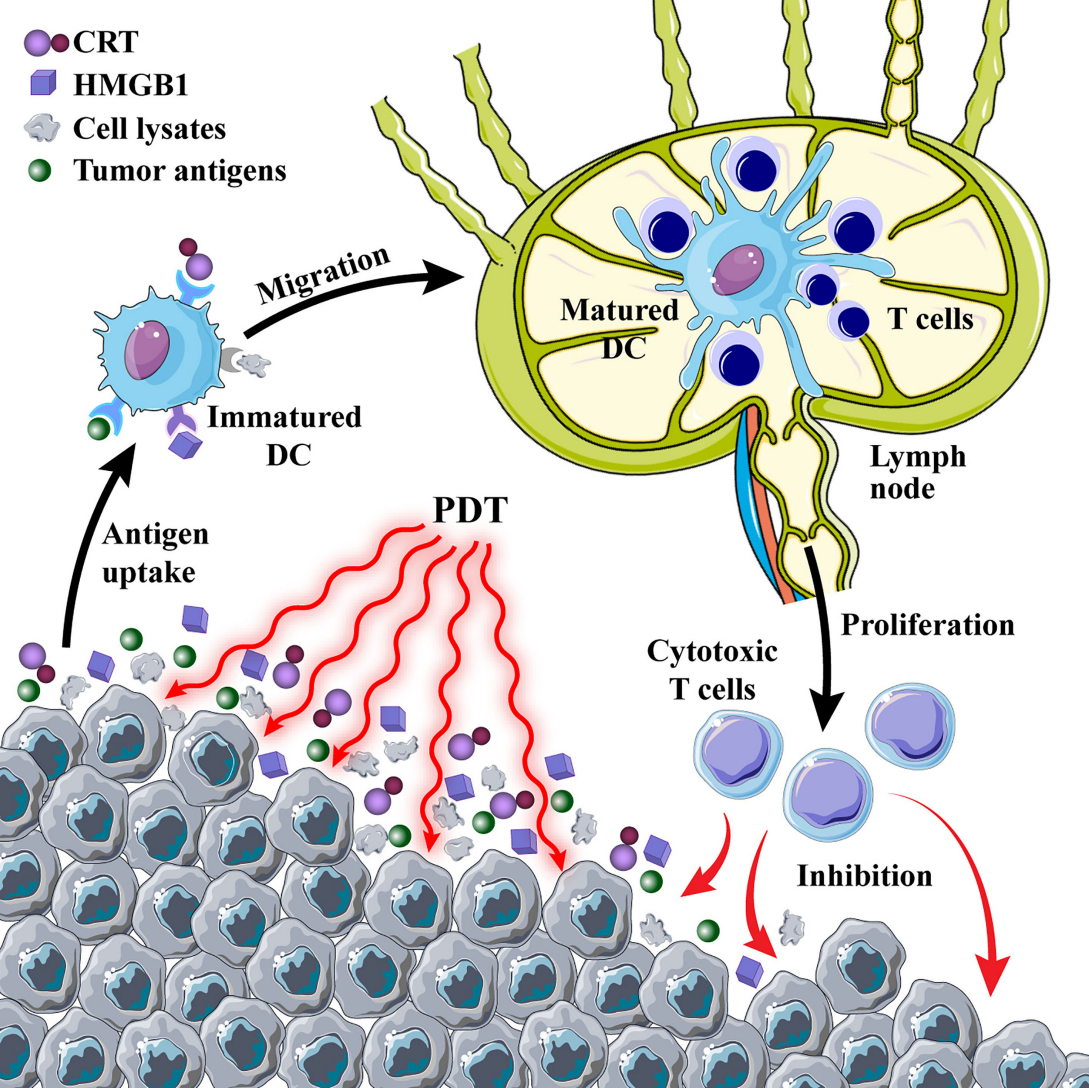
Photodynamic therapy (PDT)-induced immune response. PDT induces immunogenic cell death and promotes the release of calreticulin (CRT) and high mobility group box 1 protein (HMGB1) from tumor cells; tumor cell lysates and antigens are used as cancer vaccines to cause a series of immune cascades.

After the determination of the important role of immunogenic DAMPs in the PDT-mediated antitumor immune response, many studies have been conducted to improve the ICD triggered by PDT. Deng et al. (65) designed reduction-sensitive Ds-sP nanocarriers loaded with an efficient endoplasmic reticulum (ER)-targeting PS TCPP-T^ER^. The unique ER targeting ability of PS TCPP-T^ER^ results in an elevated level of oxidative stress in the ER of tumor cells, which in turn releases more DAMPs and enhances the immune effect. This strategy can effectively address the problems of short ROS half-life and limited intracellular diffusion depth. However, a hypoxic tumor microenvironment (TME) can limit the efficacy of PDT and reduce the efficiency of ICD induction (66). Therefore, increasing the oxygen content of tumor tissues is essential to improve the efficiency of PDT treatment. Liang and colleagues (67) developed gold nanocages (AuNCs) with hollow structures and coated them with a layer of manganese dioxide to synthesize core-shell nanoparticles (AuNC@MnO_2_). In the acidic microenvironment of tumor tissue rich in H_2_O_2_, manganese dioxide reacts as follows: MnO_2_ + H_2_O_2_ + 2H^+^→Mn^2+^ + 2H_2_O + O_2_↑ generates a large amount of oxygen to promote the accumulation of ROS in the tumor and enhances the efficacy of PDT by improving tumor hypoxia to achieve ICD. The released oxygen and Mn^2+^ can provide fluorescence (FL)/photoacoustic (PA)/magnetic resonance multimodal imaging function to evaluate the integration of tumor diagnosis and treatment. In short, the induction of ICD by enhanced PDT to promote antitumor immune response is a promising tumor treatment strategy (**Figure 3**).

### Photodynamic Therapy-Generated Cancer Vaccines

Tumor cell lysates and TAAs produced by PDT can also induce specific immune responses and are more effective than tumor cell lysates produced by ionizing radiation and ultraviolet rays (68). Similar to the inoculation mechanism of conventional vaccines that directly introduce microorganisms into the body to produce protective antibodies, cancer vaccines stimulate the activation of the body’s immune system through tumor cell death (69, 70). DCs have played a major role in the development of cancer vaccine therapy as critical mediators of antigen presentation, reversing a major component of tumor-mediated immune suppression (71, 72). Tumor residues after PDT can be used as a cancer vaccine to dramatically increase DC activation and release inflammatory cytokines to boost immune response in a mouse breast cancer model, according to a study using chlorin e6 (Ce6) as a PS (73). A promising tumor treatment strategy is to use PDT-treated tumor cells as a DC vaccine to develop a PDT-DC vaccination that can more efficiently destroy tumors and trigger a powerful antitumor immune response (74, 75).

lAntigens produced by PDT ablation of tumors may have insufficient immunogenicity as a DC vaccine and are limited to immunosuppressed “cold” tumors (76). Korbelik made a vaccine against SCCVII cells using PDT to destroy the cells and used this vaccine in the SCCVII tumor model to show that it inhibits tumors growth (77). However, the levels of splenic myeloid-derived suppressor cells (MDSCs) were significantly enhanced. Therefore, the immune adjuvant *N*-dihydrogalactochitosan (GC) was added to the PDT vaccine group to reduce the number of MDSCs (precursors of DCs, macrophages, and granulocytes) and alleviate immunosuppression (78). Ni et al. (79) used the amphipathic 4T1 breast cancer cell membrane to load PS Ce6 and the chemotherapeutic drug, i.e., doxorubicin hydrochloride (Dox), and coated the cell membrane surface with calcium carbonate to construct nanodrug delivery systems. Ce6-based PDT and Dox cause DNA damage, induce tumor ICD, and release TAA. The ROS generated during this process is expected to form *in situ* PDT-DC vaccination by mimicking inflammatory mechanisms to recruit DCs. In PDT-DC vaccinated mice, the growth of both primary 4T1 and untreated distant tumors was suppressed, indicating the establishment of an efficient immune response. Moreover, serum levels of inflammatory cytokines in mice increased continuously, peaking and then stabilizing the day after vaccination. It provides a novel antitumor combination therapy for improving the immunogenicity of the PDT-DC vaccine by introducing adjuvants or chemotherapeutic drugs. This therapy enhances body-specific immune responses, eliminates tumors, and builds long-term immunological memory.

## Combined Tumor Treatment Strategy Based on Photodynamic Therapy and Immunotherapy

### Photodynamic Therapy and Immune Adjuvants

Immune adjuvants are chemicals that boost the cellular or humoral immune response to an antigen (80, 81). Vaccines, which are one of the most successful medicinal discoveries against a variety of infectious diseases, occasionally require a molecule in conjugation to boost the immune response (82). It is therefore expedient to co-administer these with an adjuvant to ensure a high-quality/high-quantity, memory-enhanced antibody response. In chronological order of appearance, the first immune adjuvant to be used clinically was Alum, followed by the development of oil-in-water emulsions and toll-like receptor (TLR) agonists (83, 84). TLR agonists are currently being used as immune adjuvants to activate TLR signaling pathways and boost immunological responses and are found to be promising agents for cancer treatment (85, 86). This review focuses on summarizing the strategies of TLR agonists in combination with PDT.

TLRs are one of the PRRs that are expressed by a wide range of immune cells and have received more attention. To date, 13 different TLRs (TLR1–13) have been identified in mammals (87). The TLR7 agonist imiquimod (R837) is a synthetic imidazoquinoline-like molecule, approved by the US Food and Drug Administration (FDA) as a single drug and commonly used in the treatment of various skin diseases, including basal cell carcinomas (88). R837 interacts with TLR7 on the DC surface and endosomes that results in the stimulation of DC maturation and release of pro-inflammatory cytokines through elevated expression of co-stimulatory molecules (89). PDT using the PS 5-aminolevulinic acid (ALA) in combination with imiquimod cream has been proven to be useful in the treatment of squamous cell carcinoma of the skin (90). This combination therapy ameliorates the poor oncogenic effect caused by insufficient local penetration of the PS into the tumor. Because of the limitations of topical use of imiquimod cream, this agent can only be used for superficial skin cancer treatment. If tumors in internal organs of the body are to be destroyed, R837 must be delivered locally to the tumor through blood circulation. R837, being a small-molecule, is diffused after local injection, and few of them eventually reach the tumor site (91). Furthermore, R837 causes direct cell death by inducing autophagy and has concentration-dependent cytotoxicity (92–94). The use of nanomaterials to encapsulate immune adjuvants and decrease their harmful effects is a viable technique for addressing such issues. Xu et al. (95) used the hydrophobic region between UCNP and PEG to load the PS chlorin e6 (Ce6) and the TLR7 agonist R837. The results showed that TAA released from PDT-killed tumor cells and R837 induced DC maturation and released cytokines related to innate and adaptive immunity, such as TNF-α and IL-12, by upregulating the expression of co-stimulatory molecules such as CD80 and CD86. Resiquimod (R848), a second-generation derivative of R837, shares a similar structure and properties with R837. However, in contrast to R837, R848 can be used as an agonist of both TLR7 and TLR8 (96, 97). Many studies on R848 immunotherapy, both alone and in combination with chemotherapy and photothermal therapy, have been reported, suggesting that R848 can enhance immunity and improve anticancer therapeutic effectiveness (98–100). However, studies on the combined application of R848 with PDT have not been reported, which may be a promising direction for future research.

Other TLR agonists have been used in PDT immunotherapy to enhance the immune response. According to the reported study, the combination of PDT and TLR5 agonist flagellin (FlaB-Vax) effectively inhibited bilateral melanoma in mice, enhanced TME tumor antigen cross-presentation, and promoted tumor CD8^+^ T-cell infiltration and systemic IFN-γ secretion (101). CpG oligodeoxynucleotides (CpG ODN) are synthetic DNA fragments that function as TLR9 agonists by interacting with DC-expressed TLR9 and enhancing antigen-specific immune responses (102). Ni and co-workers (103) used the cationic PS 5,10,15,20-tetra(*p*-benzoato)porphyrin (TBP) to adsorb the anion CpG to achieve efficient PDT and effective delivery of CpG. In a mouse breast cancer model, the combination of PDT and CpG was found to provide excellent tumor suppression, with about 97% of tumors being eliminated. Cai et al. (104) designed a metal–organic framework (MOF) nanoparticle formed by the self-assembly of the PS H_2_TCPP and zirconium ions. The porous internal structure of MOF was used to load the TLR9 agonist CpG ODN. When compared with the control and the treatment group alone, the combination of CpG ODN and PDT dramatically increased the expression of MHC-II, CD317, and co-stimulatory molecules including CD80/CD86. Remarkably, CpG ODN also reduced the immunosuppressive activity of MDSCs and improved the tumor immunosuppressive microenvironment (105). However, CpG ODN is still mostly used as an immunostimulant in PDT immunotherapy, and there is a significant research gap in improving immunosuppression.

Many immune adjuvants such as glycated chitosan (GC), lactobacillus BCG, mycobacterial cell wall extract (MCWE), complete Freund (CF) adjuvant, and incomplete Freund (IF) adjuvant can stimulate the immune response similar to that of TLR agonists (106). Previously, it was demonstrated that GC, a water-soluble compound synthesized from galactose and chitosan, was shown to stimulate TNF-α secretion by macrophages and induce tumor-specific immune responses (107). Cai’s group (108) found the highest levels of apoptotic and inflammatory responses and the highest infiltration of immune cells in tumors in the synergistic treatment group of PDT and GC. Additionally, mice treated with a combination of PDT and GC had a significantly better survival rate in the EMT6 mammary tumor and 4T1 metastatic mammary tumor models. In short, PDT adjuvant by immune adjuvant has promising research potential because of its ability to inhibit tumor metastasis and recurrence.

### Photodynamic Therapy and Inhibitors of Immune Suppression

**Figure 4:**
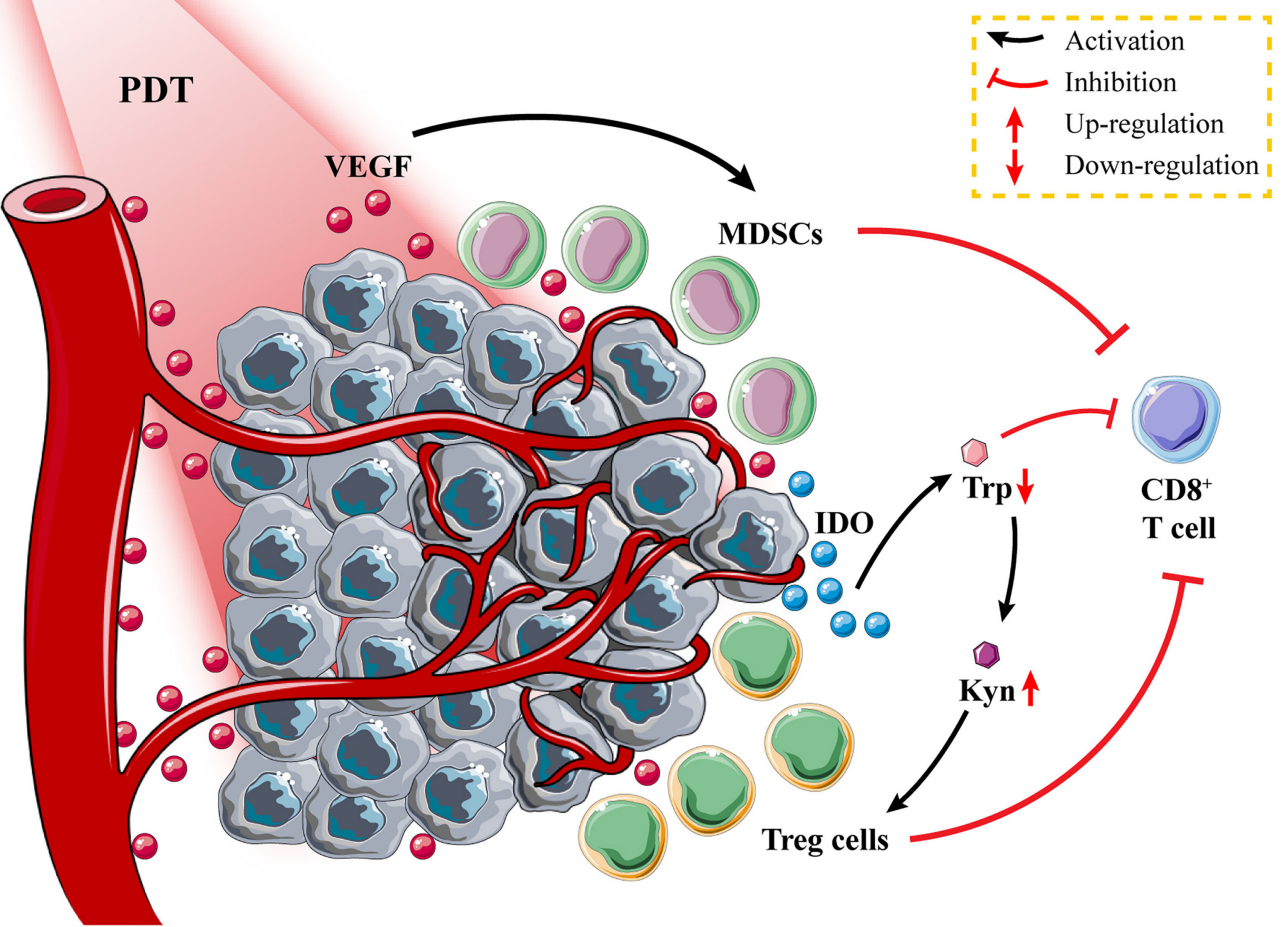
Tumor immunosuppressive environment after photodynamic therapy (PDT). Tumor tissues secrete vascular endothelial growth factor (VEGF) to promote the proliferation of myeloid-derived suppressor cells (MDSCs); the high expression of indoleamine 2,3-dioxygenase (IDO) in tumor cells promotes the recruitment of Treg cells and inhibits the activation of CD8^+^ T cells through tryptophan metabolism.

PDT can activate the immune system to some extent, but the intensity of the PDT-induced immune response may not be sufficient to destroy the tumors or prevent their metastasis and recurrence due to the immunosuppressive effect of the TME and immune escape of tumor cells (109, 110) (**Figure 4**). Moreover, PDT causes local inflammation and immunosuppression due to contact hypersensitivity (CHS) (111). Therefore, it is necessary to explore suitable immune inhibitors to inhibit tumor immunosuppressive signals and enhance PDT-induced immune responses.

Immunosuppressive cells such as MDSCs and regulatory T cells (Tregs) in the TME suppress the antitumor immune responses and promote tumor progression and invasion (112, 113). Specifically, MDSCs suppress T-cell function through multiple mechanisms, including production of nitric oxide and immunosuppressive metabolites, secretion of immunosuppressive cytokines such as TGF-β and IL-10, and upregulation of cyclo-oxygenase 2 (Cox2) and prostaglandin E2 (PGE2) (114, 115), while Tregs suppress T-cell function through CTL antigen 4 to inhibit the expression of DC co-stimulatory molecules including CD80 and CD86 (116). Recently, it has been found that PDT vaccination significantly increased MDSC and Treg levels, while low doses of GC and cyclophosphamide were found to reduce the elevated levels of immunosuppressive cells (77, 117). The findings demonstrated the feasibility of using small-molecule inhibitors to weaken immunosuppressive cells and alter the immunosuppressive TME, thereby enhancing the PDT-DC vaccine-induced immune responses.

Apart from their role in tumor development and immune escape, the reprogramming of tumor cells’ metabolism influences immune cell metabolism (118). Tryptophan (Trp) metabolism in T cells is mediated by tumor cells through an elevated level of indoleamine 2,3-dioxygenase (IDO) expression, which converts Trp to kynurenine (Kyn) (119). Lack of Trp inhibits CTL activation, while abnormal accumulation of Kyn recruits Treg to suppress effector T-cell function (120, 121). Reducing the level of immunosuppression by PDT nanoparticles loaded with IDO inhibitors is a more appropriate strategy. Zhao et al. (122) constructed self-delivery photo-immune stimulators (iPSs) through non-covalent interactions between the PS Ce6 and IDO inhibitor, i.e., NLG919. They first demonstrated that iPSs can promote DC maturation by inducing apoptosis and ICD in CT26 cells *via* PDT and cellular release of CRT and HMGB1. Following PDT treatment with iPSs, increased CD4^+^/CD8^+^ T-cell infiltration was observed in the mouse CT26 tumor tissues, while a significant decrease in Kyn/Trp ratio was observed in the serum. Additionally, transcriptomic analysis of mouse tumor tissues demonstrated that iPS-based PDT could effectively stimulate the tumor immune microenvironment and enhance tumor immunotherapy efficacy. A variety of PSs and IDO inhibitors, such as self-assembled nanoparticles, are being developed extensively. Yang’s group (123) designed pH-responsive nanovesicles (pRNVs) as carriers to synthesize PRNVS/HPPH/IND smart nanoparticles by encapsulating the PS HPPH and the IDO inhibitor indoximod (IND) through hydrophobic interactions. They found that PDT treatment of the nanoparticles inhibits mouse melanoma growth, and the release of IND stimulates CD8^+^ T cells to destroy distant tumors by increasing P-S6K phosphorylation.

Tumor tissues secrete large amounts of vascular endothelial growth factor (VEGF) to promote the proliferation of immunosuppressive cells and inhibit DC maturation by NF-κB pathway activation (124, 125). Excess VEGF leads to abnormalities in tumor vascular structure and function, exacerbating the hypoxic state of the TME and affecting the efficacy of PDT therapy (126). Zhou et al. (127) developed a self-assembled nanoplatform containing the PS Ce6, the VEGF receptor (VEGFR) inhibitor axitinib (AXT), and the IDO inhibitor dextro-1-methyl tryptophan (1MT) to alleviate immunosuppression by promoting vascular normalization and improving the tumor hypoxic microenvironment, thereby enhancing PDT immunotherapy. According to the obtained results, the enhanced PDT immunotherapy has significant effects on both primary melanoma and lung metastases in mice.

The PS-based PDT induces tumor ICD and stimulates immune activation, while the inhibitor of immune suppression promotes PDT-induced immune response by weakening tumor immune escape (128). It is about the immunosuppressive TME of MDSCs and Tregs and immunosuppressive molecules like IDO-1 and VEGF. PDT should be combined with compounds that can inhibit MDSCs and/or Tregs and IDO or VEGF.

### Photodynamic Therapy and Immune Checkpoint Blockade

Various immunosuppressive mechanisms can impair the efficiency of antitumor immunotherapy during tumor progression. For example, immune checkpoint molecules are considered to be the primary anticancer immunotherapy targets, as they have a negative immunomodulatory effect (129). The development of target-specific antibodies to block the underlined immune checkpoints is a hot topic in immunotherapy. However, due to the low tumor immunogenicity, the response rate of some patients to ICB therapy is unsatisfactory (130). The efficiency of ICB therapy can be improved by enhancing tumor immunogenicity and sensitivity through PDT-mediated ICD induction (131, 132). Herein, three immune checkpoints, including CTLA-4, PD-1/PD-L1, and CD47, have been described, followed by summarizing their combined treatment strategies along with PDT.

#### CTLA-4 Checkpoint Blockade

CTLA-4 is an immune checkpoint receptor expressed on Tregs and other activated T cells (133). It binds CD80 and CD86 ligands on DCs, weakening T-cell activation and inability to perform normal immune functions (134). It is considered the first immune checkpoint receptor to be used clinically for cancer immunotherapy. Additionally, ipilimumab, an anti-CTLA-4 monoclonal antibody used to treat metastatic melanoma, has been approved by the FDA (135). The combination of anti-CTLA-4 antibody and PDT is essential for the eradication of systemic tumors and may be an effective therapeutic strategy for advanced cancers (136). Wang et al. (137) developed bullet-shaped magnetic mesoporous organosilica nanoparticles (M-MONs) with Fe_3_O_4_ at the head and a mesoporous silica framework at the tail. Next, the redox/pH dual-responsive M-MONs@Ce6 nanoparticles were developed with M-MONs (pores size of ~3.8 nm) and loaded with the PS Ce6. M-MONs@Ce6 induced more severe ICD by simultaneous PDT and magnetothermal treatment under the combined action of laser and alternating-current magnetic field (ACMF), releasing DAMPs to trigger specific immune responses and significantly inhibiting the growth of mouse and human breast cancers. In a mouse model of breast cancer with lung metastasis, the PDT+anti-CTLA-4 antibody treatment group and the magnetothermal treatment+anti-CTLA-4 antibody treatment group showed an inhibitory effect on lung metastatic tumor. This inhibitory effect was further enhanced when the two treatments were combined, accompanied by an increase in CTL and a decrease in Treg. Furthermore, the nanoparticles used for PDT treatment had no severe side effects when combined with ICB treatment, indicating that this is a safe and effective strategy for the treatment of metastatic cancer.

#### PD-1 and PD-L1 Checkpoint Blockade

Following the success of CTLA-4 checkpoint blockade in antitumor immunotherapy, more consideration has been paid to the exploration of new immune checkpoints. PD-1, also known as CD279, is an immunosuppressive signaling molecule highly expressed on tumor-specific T cells. PD-1 binds to PD-L1 (PD-1 ligand) present on the tumor cells. Consequently, the inhibition of T-cell proliferation and activation, elevated levels of T-cell apoptosis, and reduced cytokine secretion were observed (138, 139). PD-1/PD-L1 signaling promotes tumor immune escape and severely affects the efficacy of cancer immunotherapy (140). For this reason, several anti-PD-1/PD-L1 antibodies have been developed for restoring T-cell viability and promoting antitumor immune response (141). It has been reported that PDT significantly increases tumor PD-L1 levels, while the majority of recruited CD8^+^ T cells express PD-1, emphasizing the importance of a combined anti-PD-1/PD-L1 antibody therapeutic strategy (142, 143). Liu et al. (144) used PS-g-PEG micelles to encapsulate the PS, i.e., BDP-I-N to improve its water solubility, followed by modifying the micelles with functional groups to attach anti-PD-L1 antibodies that result in the synthesis of BDP-I-N-anti-PD-L1 multifunctional nanoparticles. Unlike the conventional therapeutic strategy to achieve the combination with PDT by intravenous injection of anti-PD-1/PD-L1 antibodies, this work assembled anti-PD-L1 antibodies and PSs into nanoparticles and accomplished the efficient accumulation in tumor tissues through the active targeting of immune checkpoint antibodies and the EPR effect of nanoparticles. *In vivo* results demonstrate that BDP-I-N-anti-PD-L1 nanoparticles eliminate MC38 mouse colon tumors by synergistic action of PDT and ICB, generate immune memory to prevent tumor recurrence, and have an excellent biosafety profile.

Anti-PD-1/PD-L1 antibodies have demonstrated excellent efficacy in tumor immunotherapy; however, the high cost of these antibodies adds to the financial burden of cancer patients (145). To address the high cost of anti-PD-L1 antibodies, Zhang and colleagues (146) extracted PD-1-expressing HEK293T cell membranes to replace anti-PD-L1 antibodies to bind PD-L1 on 4T1 cells. They developed PDT-mediated PFTBA@HSA-DVMS (PHD) nanoemulsions against hypoxic tumors by wrapping the oxygen supply agent perfluorotributylamine (PFTBA) in human serum albumin (HSA), followed by loading it with the PS sinoporphyrin sodium (DVDMS). In addition, they developed PHD@PM nanoplatform by encapsulating PHD nanoemulsion inside PD-1-expressing cell membranes to realize the combination of PDT and ICB. Their work demonstrates that PHD@PM nanoplatform is an innovative therapeutic platform. This platform has high clinical application because of its low cost, high biocompatibility, and active targeting. Furthermore, it has the ability to improve TME, which is hypoxic and immunosuppressive.

#### CD47 Checkpoint Blockade

Aside from the two most widely studied immune checkpoints mentioned above, one of the current hot spots in cancer ICB therapy is targeting the CD47–SIRP signaling axis. CD47 is a membrane protein expressed by almost all cells, while signal-regulating protein α (SIRPα) is only expressed by myeloid cells such as macrophages and monocytes (147, 148). CD47 on the surface of tumor cells binds to SIRPα on macrophages releasing a “don’t eat me” signal that inhibits macrophage phagocytosis and thus promotes tumor immune escape (149). A reported study has revealed that thrombospondin-1 (TSP-1) in the TSP-1/CD47/SIRP-α signal axis could significantly improve treatment outcomes by blocking it, laying the groundwork for the clinical use of PDT cancer vaccines (150). Chang et al. (151) designed Cu_2_O@CaCO_3_ nanoparticles for the target-specific treatment of colorectal cancer (CRC). In the acidic microenvironment of CRC, the CaCO_3_ shell decomposed to release the PS precursor Cu_2_O. Exposed Cu_2_O reacted with endogenous H_2_S in the CRC, producing Cu_31_S_16_ and ROS to achieve PDT under 1,064-nm laser radiation. It was found that the oxidative stress induced by Cu_2_O@CaCO_3_ nanoparticles could also promote macrophages from immunosuppressed M2 phenotype to immune-activated M1 phenotype. Combined treatment with anti-CD47 antibody resulted in improved phagocytosis of macrophages, promoted antigen presentation, and induced antitumor immune response by T cells, achieving effective inhibition of CRC metastasis and recurrence. Furthermore, recent studies have also indicated that the combination of PDT and CD47 monoclonal antibodies may have the potential for the treatment of human bladder cancer (152).

The combination of PDT and ICB can effectively inhibit tumor metastasis and recurrence compared with the individual therapeutic effect of PDT. Moreover, the combination strategy also improves the failure of ICB treatment due to insufficient immunogenicity of tumor cells. Current studies have identified several new immune checkpoints, such as V-domain Ig suppressor of T-cell activation (VISTA), T-cell immunoglobulin and ITIM domain (TIGIT), and T-cell immunoglobulin and mucin-domain containing-3 (TIM-3) (153, 154). Taken together, the combination of immune checkpoints and PDT may be a promising direction for future research.

**Figure 5:**
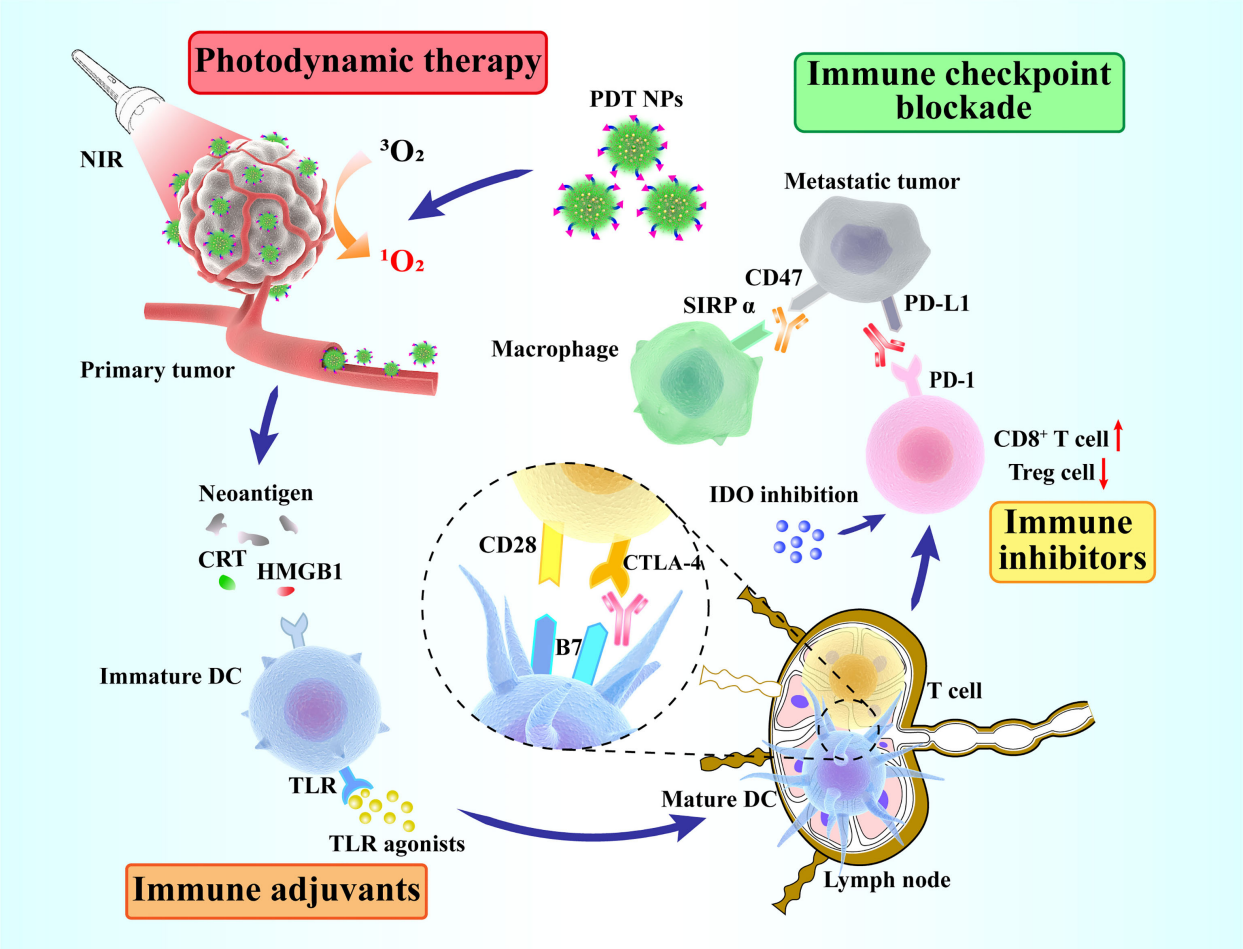
Schematic overview of synergized photodynamic immunotherapy. Photodynamic therapy enhances the antitumor immune response, thus killing primary and distant tumors in combination with different immunotherapeutic strategies (immune adjuvants, immune inhibitors and immune checkpoint blockade).

**Figure 5** shows the combined treatment strategies of different types of PDT immunotherapy. These strategies exert significant effects in cancer treatment mainly by enhancing the anticancer immune response or by reducing the suppression of the immune system. The specific details of the combination of PDT and different types of immunotherapies are summarized in **Table 1**.

**Table 1:** Summary of photodynamic therapy synergized immunotherapy.

Combination strategies	Photosensitizers/λ _ex_ (nm)	Immunotherapy reagents	Tumor models	Effector cells and cytokines	Ref
PDT and immune adjuvants	5-Aminolevulinic acid/630	TLR7 agonist (imiquimod)	SCC invasive squamous cell carcinoma	CD4^+^ and CD8^+^ T cells IFN-α, TNF-α, IL-6, IL-8, CXCL9 and CXCL10	(90)
	UCNP-Ce6/980	Imiquimod (R837)	CT26 mouse colon adenocarcinoma	DCs, CD8^+^ T cells, Treg T_CM_ and T_EM_ IL-12p40, IFN-γ, TNF-α	(95)
	Pheophorbide A/671	TLR5 agonist (FlaB-Vax)	B16-F10 mouse melanoma	memory CD8^+^ T cells CD103^+^ DCs, IFN-γ	(101)
	W-TBP/650	TLR9 agonist (CpG)	TUBO murine breast adenocarcinoma	T cells, NK cells, DCs macrophages, IFN-α, IL-6	(103)
	H_2_TCPP/670	TLR9 agonist (CpG)	H22 mouse hepatocellular carcinoma	DCs, CD4^+^/CD8^+^ T cells TNF-α, IFN-γ, IL-12p70	(104)
	Photofrin/630	Glycated chitosan	EMT6 and 4T1 murine breast carcinoma	CD3^+^/CD8^+^ T cells	(108)
PDT and immune inhibitors	Chlorin e6/630	IDO-1 inhibitor (NLG919)	CT26 murine colorectal cancer	DCs, CD4^+^/CD8^+^ T cells Treg, Kyn/Trp	(122)
	HPPH/671	IDO inhibitor Indoximod (IND)	B16F10 mouse melanoma	DCs, CD8^+^/CD4^+^ T cells IL-6 and TNF-α	(123)
	Chlorin e6/660	VEGFR inhibitor Axitinib (AXT)	B16F10 mouse melanoma	T cells, TAM IL-2, IL-6, IFN-γ	(127)
PDT and immune checkpoint blockades	Bremachlorin/662	Anti-CTLA-4 antibody	MC38 and CT26 colorectal cancer	CD8^+^ T cells CD4^+^ regulatory T cells	(136)
	Chlorin e6/660	Anti-CTLA-4 antibody	MCF-7 and 4T1 breast carcinoma	DCs, CTLs, Treg TNF-α, IFN-γ, IL-6	(137)
	BDP-I-N 740 or 808	Anti-PD-L1 antibody	MC38 murine colorectal cancer	—	(144)
	Sinoporphyrin sodium/635	PD-1 protein	4T1 mouse breast carcinoma	DCs, CTLs, T_h_ cells, Treg TNF-α, IL-10	(146)
	Cu_2_O/Cu_31_S_16_ 1064	Anti-CD47 antibody	CT26 murine colorectal cancer	M2/M1 TAM, CTLs IL-10, IL-12	(151)

PDT, photodynamic therapy; DCs, dendritic cells; IDO, indoleamine 2,3-dioxygenase; CTL, cytotoxic T lymphocyte.

## Conclusion

In this review, we have explained the classification and photophysical mechanism of PDT based on the Jablonski Diagram. By inducing ICD, PDT has been shown to successfully activate the immune response. However, due to insufficient immunogenicity or immunosuppression, the immune reaction induced by a single PDT is greatly restricted. Therefore, PDT with other immunotherapies has been integrated to solve such problems. However, optimizing the *in vivo* safety assessment is still a challenge and needs further research to enhance the efficiency of PDT immunotherapy for effective tumor treatment.

Immunotherapy has been extensively studied in clinical trials, but clinical studies on PDT and its effects on the human immune system are very rare. Although a large number of PSs have been developed and used for PDT in animal studies, Photofrin and aminolevulinic acid (ALA) are the few two used in clinical research. And only a few of these studies have investigated on the effect of PDT on the human immune system (155, 156). Determining the relationship between PDT and immune response in clinical research and combining it with immunotherapy will be a major focus for future research. We hope that PDT immunotherapy can be proven to be an excellent cancer treatment in clinical trials.

## Author Contributions

YH contributed to the conception and design of the review. JHu and PW wrote the manuscript. LG, ZZ, JHe, LZ, and YZ revised the manuscript. All authors contributed to the article and approved the submitted version.

## Funding

This work was supported by the National Natural Science Foundation of China (No. 82072340), the Major national science and technology projects-Major new drug creation (2019ZX09301-132), Changjiang Scholars and Innovative Research Team in University (No. IRT_15R13), and Guangxi Science and Technology Base and Talent Special Project (No. AD17129003).

## Conflict of Interest

The authors declare that the research was conducted in the absence of any commercial or financial relationships that could be construed as a potential conflict of interest.

## Publisher’s Note

All claims expressed in this article are solely those of the authors and do not necessarily represent those of their affiliated organizations, or those of the publisher, the editors and the reviewers. Any product that may be evaluated in this article, or claim that may be made by its manufacturer, is not guaranteed or endorsed by the publisher.
